# Thermally Stable and Antimicrobial Active Poly(Catechin) Obtained by Reaction with a Cross-Linking Agent

**DOI:** 10.3390/biom11010050

**Published:** 2020-12-31

**Authors:** Malgorzata Latos-Brozio, Anna Masek, Małgorzata Piotrowska

**Affiliations:** 1 Faculty of Chemistry, Institute of Polymer and Dye Technology, Lodz University of Technology, Stefanowskiego 12/16, 90-924 Lodz, Poland; malgorzata.latos@p.lodz.pl; 2 Faculty of Biotechnology and Food Sciences, Institute of Fermentation Technology and Microbiology, Lodz University of Technology, Wólczańska 71/173, 90-924 Lodz, Poland; malgorzata.piotrowska@p.lodz.pl

**Keywords:** (+)-catechin, polymerization, cross-linking compound, antioxidant properties, thermal analysis

## Abstract

(+)-Catechin is a flavonoid with valuable antioxidant and antimicrobial properties, found in significant amounts in green tea leaves. Polymeric forms of catechin have been obtained by enzymatic reaction, photopolymerization, and polycondensation in designed processes. However, so far, poly(catechin) has not been received in the cross-linking reaction. Reactions with the cross-linking compound allowed for the preparation of antibacterial and antioxidant materials based on quercetin and rutin. The aim of the research was to obtain, for the first time, poly(catechin) by reaction with glycerol diglycide ether cross-linking compound. The polymeric form of (+)-catechin was confirmed using FTIR and UV-Vis spectroscopy. In addition, thermal analysis (TG and DSC) of the polymeric catechin was performed. The antioxidant and antibacterial activity of poly (flavonoid) was also analyzed. Poly(catechin) was characterized by greater resistance to oxidation, better thermal stability and the ability to reduce transition metal ions than (+)-catechin. In addition, the polymeric catechin had an antimicrobial activity against *Staphylococcus aureus* stronger than the monomer, and an antifungal activity against *Aspergillus niger* comparable to that of (+)-catechin. The material made on the basis of (+)-catechin can potentially be used as a pro-ecological stabilizer and functional additive, e.g., for polymeric materials as well as dressing materials in medicine.

## 1. Introduction

Catechin (flavan-3-ol) and its derivatives are polyphenolic compounds, found in significant amounts in green tea, cocoa, red wines, and chocolate. This plant flavonoid is known for its strong antioxidant properties and has been proposed in the literature as an anti-aging substance. Moreover, catechins also show antibacterial and antifungal activity [1,2,3,4,5,6].

The valuable properties of compounds from the group of flavonoids closely depend on their chemical structure. Flavonoids are based on the flavan structure, thus the number, positions, and types of substitutions influence radical scavenging and chelating activity, as well as their pharmacological activities such as antiviral/antibacterial, cytotoxic, cardioprotective and anti-inflammatory activities. The relationship of the structure and properties of low molecular weight flavonoids is analyzed in detail. The influence of individual structural elements of flavonoids on the efficiency of scavenging free radicals and pharmacological action is described comprehensively. However, correlations between the structure of polymeric forms and their activity are poorly researched [7,8,9,10,11].

In plant materials, catechins occur in many oligomeric forms, primarily condensed tannins (also known as proanthocyanidins) [12]. Literature data show that the polymerized structures of flavonoids may be characterized by stronger antioxidant properties, better antimicrobial activity and higher thermal stability [13,14,15]. It has been shown that increasing the degree of polymerization of oligomeric or polymeric flavan-3-ols increases their radical reduction efficiency. Significant free radical scavenging properties by the polymeric structures are the result of extensive conjugation between 3-OH and B-ring catechol groups, together with abundant β4→8 linkages [16]. Moreover, studies have shown that dimeric flavan-3-ols play an important, protective role in the human diet [17].

As far as this, the polymeric catechin has been obtained by enzymatic polymerization [18,19,20], photopolymerization [21], HCl acid catalyzed polymerization [22], and polycondensation of catechins with aldehydes in the presence of acid catalyst [23,24,25].

Another method to acquire poly(flavonoids) is a polymerization reaction with a crosslinking compound. Sahiner proposed a method of obtaining polymeric forms of flavonoids, such as quercetin and its glycoside - rutin, consisting in polymerization with a cross-linking compound in the form of glycerol diglycide ether (GDE), using L-α lecithin as a surface-active agent, in a cyclohexane environment in the case of obtaining poly(quercetin) or in the gasoline environment when obtaining poly(rutin). Polyfunctional crosslinker glycerol diglycidyl ether (GDE) is a biocompatible material and can be used to connect monomeric flavonoids into a particle form. The epoxy groups in GDE readily react with the phenolic OH groups in the flavonoids to form polymeric structures [26,27].

The aim of the study is to obtain poly(catechin) by reaction with an epoxy cross-linking agent. Based on the literature data review, it was found that this polymeric, cross-linked catechin compound has not yet been described. The crosslinking reaction was used only to prepare poly(quercetin) and poly(rutin) [26,27]. The molecule of quercetin and rutin, flavonoids from the flavonols group, contains, like most flavonoids, a carbon skeleton with a ketone group in the 4-position, while catechins from the flavan-3-ol group of flavonoids do not contain a keto group in the carbon skeleton, and so they are fundamentally different in chemical structure from quercetin and rutin. Besides the polymerization reaction of catechin with the crosslinker, this manuscript also describes the properties of the obtained poly(catechin), such as antioxidant activity, thermal stability, and antibacterial properties

## 2. Materials and Methods

### 2.1. Preparation of Poly(Catechin) in the Cross-Linking Reaction

Catechin ((+)-catechin hydrat ≥98% HPLC, MW: 290,27 g/mol, Sigma Aldrich, product of China) polymerization was performed according to the method proposed by Sahiner [26,27] with minor modifications. First, a solution of (+)-catechin was prepared by dissolving 1 g of (+)-catechin in 10 mL of 1 M NaOH (ChemPur, Piekary Slaskie, Poland). Then, 4 mL of this solution was added to 150 mL of a 0.1 M solution of L-α-lecithin (from soybean, ≥99%, MilliporeSigma, Darmstadt, Germany) in cyclohexane (96%, pure. P.A., ChemPur, Poland). The solution was stirred for 2 h at 1000 rpm at 20 °C, after which time glycerol diglycidyl ether (GDE, technical grade, Sigma-Aldrich, Steinheim, Germany) was added in an amount of 100 mol% with respect to the catechin used. After 2 h of stirring (1000 rpm), the obtained poly(catechin) was washed twice with cyclohexane by centrifugation (6000 rpm, room temperature). The poly(catechin) was dried at 35 °C for 72 h.

### 2.2. Infrared (FTIR) and UV-Vis Spectroscopy

A Nicolet 670 FTIR spectrophotometer (Thermo Fisher Scientific, Waltham, MA, USA) was utilized to analyze the poly(catechin) structure. Samples of (+)-catechin and polymeric form of (+)-catechin were placed at the output of infrared beams. Oscillating spectra were obtained as the result of the test. The analysis of oscillating spectra allows determination of the functional groups with which the radiation interacted.

The spectra of (+)-catechin and poly(catechin) powders at wavelengths of 190–1100nm were recorded using a UV-Vis spectrophotometer (Evolution 220, Thermo Fisher Scientific, Waltham, MA, USA).

### 2.3. Scanning Electron Microscopy (SEM)

Based on the images obtained from the scanning electron microscope (SEM) LEO 1530 (Carl Zeiss AG, Oberchoken, Germany), the morphology of (+)-catechin and poly(catechin) powders was evaluated. Magnification was 10,000, 25,000 and 50,000×.

### 2.4. Thermal Analysis of (+)-Catechin and Poly(Catechin)

Thermogravimetric (TG) analysis: thermal stability of (+)-catechin and poly(catechin) was performed utilizing a Mettler Toledo Thermobalance (TA Instruments, Greifensee, Zürich, Switzerland). Samples of 10 mg were placed in alumina crucibles and heated from 25°C to 800°C under argon flow (50 mL/min) at heating rate of 5 °C/min.

Differential scanning calorimetry (DSC): temperature ranges of (+)-catechin and poly(catechin) phase changes were determined using a Mettler Toledo DSC analyser (TA 2920; TA Instruments, Greifensee, Zürich, Switzerland). The samples of 5–6 mg (placed in 100 μL aluminium pans) were heated from −80 to 400 °C at a rate of 10 °C/min in air.

For thermal analysis (DSC and TGA) the values specified by the apparatus manufacturer were given as the measurement uncertainty.

### 2.5. Antioxidant Tests

ABTS and DPPHaAnalysis: The antioxidant capacity of catechin and poly(catechin) was determined by ABTS and DPPH tests. These methods are based on reduction of free radicals ABTS (2,2′-azino-bis(3-ethylbenzothiazoline-6-sulphonic acid)) and DPPH (2,2-diphenyl-1-picrylhydrazyl).

The ABTS^•+^ radical was obtained by the mixing of a 6 mM ABTS (assay ≥ 98%, Sigma Aldrich, Saint Louis, MO, USA) solution in distilled water with potassium persulfate (2.45 mM; 99.99%, Sigma Aldrich, Saint Louis, MO, USA) in the dark at 20 °C for 15 h before use. The absorbance of the ABTS^•+^ solution was adjusted with EtOH (pure P.A., 96%, POCH, Gliwice, Poland) to 0.70 ± 0.03 at 734 nm at room temperature. Then, the ABTS^•+^ solution (1.0 mL) was mixed with 0.95 mL of ethanol and 50 μL of catechin or poly(catechin) (0.1 mg/mL in distilled water). The absorbance was measured at 734 nm after 2 min utilizing a UV-spectrophotometer (Evolution 220, Thermo Fisher Scientific, Waltham, MA, USA).

The ethanolic solution of DPPH (2.0 mL, 40 mg/mL; <=100%, Sigma Aldrich, Germany) was added to 0.5 mL of distilled water containing 1mg/mL catechin or poly(catechin)-DPPH solution, which has a purple colour with a maximum absorbance at 517 nm. Distilled water was used as a blank in ABTS and DPPH methods.

Level of inhibition (%) of free radicals ABTS and DPPH was calculated according to the equation:

Inhibition (%) = [((A_0_ − A_1_)/A_0_) × 100 (1)

where A_0_ is the absorbance of the reference sample without antioxidants, and A_1_ is the absorbance in the presence of catechin or poly(catechin) [28].

The inhibition level (%) of absorbance was calculated using the standard curve prepared with Trolox (% inhibition level - μM Trolox). The effect of catechin and poly(catechin) on scavenging ABTS and DPPH is referred to as the Trolox equivalent antioxidant capacity (TEAC).

The ABTS and DPPH tests were performed on three control samples and the average results were shown in the manuscript. Calculations were made for the means and standard deviations of three independent samples (*n* = 3). The measurement uncertainty was given as standard deviation.

FRAP and CUPRAC Analysis: The FRAP (ferric reducing antioxidant power) and CUPRAC (cupric reducing antioxidant capacity) tests were used to assess the ability of catechin or poly(catechin) to reduce transition metal ions. The FRAP method is based on the reduction of the ferric ion (Fe^3+^→Fe^2+^) under acidic conditions. The CUPRAC test is analogous to the FRAP assay and consists of the reduction of Cu^2+^ to Cu^1+^.

The FRAP solution was freshly prepared by mixing 25 mL of acetate buffer solution (0.3 M, pH 3.6; Chempur, Piekary Slaskie, Poland), 2.25 mL of 10 mM TPTZ (2,4,6-Tris (2-pyridyl)-s-triazine; ≥99.0%, HPLC, Sigma Aldrich, Switzerland) dilution in 40 mM hydrogen chloride solution (Chempur, Piekary Slaskie, Poland) and 2.25 mL of 20 mM FeCl_3_ (pure P.A., Chempur, Piekary Slaskie, Poland) in distilled water solution. The mixture was stirred and incubated at 37 °C for 25 min. Then, the absorbance of the ferrous form with blue colour (Fe^2+^-TPTZ complex) was measured at 595 nm, utilizing a UV-spectrophotometer.

In CUPRAC method approximately 0.25 mL of CuCl_2_ (0.01 M, Chempur, Piekary Slaskie, Poland) was mixed with 0.25 mL of an ethanol solution of 7.5 × 10^−3^ M neocuproine (assay ≥98%, Sigma Aldrich, Shanghai, China) and 0.25 mL of CH_3_COONH_4_ buffer solution (1 M, Chempur, Piekary Slaskie, Poland), in a test tube followed by addition of catechin and poly(catechin) solution. The total volume of samples was increased to 2 mL with pure water. The absorbance at 450 nm was measured against distilled water as reagent blank, after 30 min incubation at room temperature.

The ferric (FRAP) and cupric (CUPRAC) ions reducing capacity was calculated according to the formula:

ΔA = A_AR_ − A_0_ (2)

where A_0_ - absorbance of the reagent sample, A_AR_ - absorbance after reaction [29].

The FRAP and CUPRAC analysis were made on three control samples and the average results were described in the manuscript. Computations were done for the means and standard deviations of three independent samples (*n* = 3). The measurement uncertainty was given as standard deviation.

### 2.6. Antibacterial and Anti-Fungal Tests

The research was carried out using the dynamic "flask shake methods". The following bacterial test strains were used in the research: *Escherichia coli* ATCC 8739, *Staphylococcus aureus* ATCC 6538, *Bacillus subtilis* ATCC 6633 and fungi: *Candida albicans* ATCC 10231 and *Aspergillus niger* ATCC 16404. The cultures were stored on slants with Merck's TSA (bacteria) and MEA (fungi) medium at 6 °C. The strains were activated before the experiment. 10 mg of catechin and poly(catechin) were placed in test tubes, to which were added 9.9 mL of nutrient broth, and then 0.1 mL of a suspension of test microorganisms suspended in physiological saline.

The samples were incubated under dynamic conditions, on a shaker (150 rpm) for 24 h at the temperature of 30 °C (*B. subtilis* and *A. niger*) and 37 °C (other strains).

The number of microorganisms in the tubes after 24 h of incubation was determined by the culture method on TSA (bacteria) and MEA (fungi). In addition, the control samples (only microorganisms) were counted at the start of the experiment (t = 0). The results are given as the number of colony forming units/mL of medium (cfu/mL). The dieback rates of microorganisms D were determined (3):

D = (log number of microorganisms _t = 0_ − log number of microorganisms _t = 24_) (3)

The antibacterial and anti-fungal tests were performed on three control samples. Averaged results were included in the manuscript. Calculations were done for the means and standard deviations of three independent samples (*n* = 3). The measurement uncertainty was given as standard deviation. The measurement uncertainty of the samples was given as standard deviation. For dieback rates of microorganisms D, no standard deviation was given because the value of D is the difference between the log number of microorganisms _t = 0_ and the log number of microorganisms _t = 24_.

## 3. Results and Discussion

Glycerol diglycidyl ether (GDE) is an aliphatic epoxy monomer that can be used as a diepoxy crosslinker. Its properties include low shrinkage, good adhesion, and good thermo-mechanical properties. GDE can be used in the formation of epoxy materials, which can further be used in biodegradable plastics [30,31].

**Figure 1:**
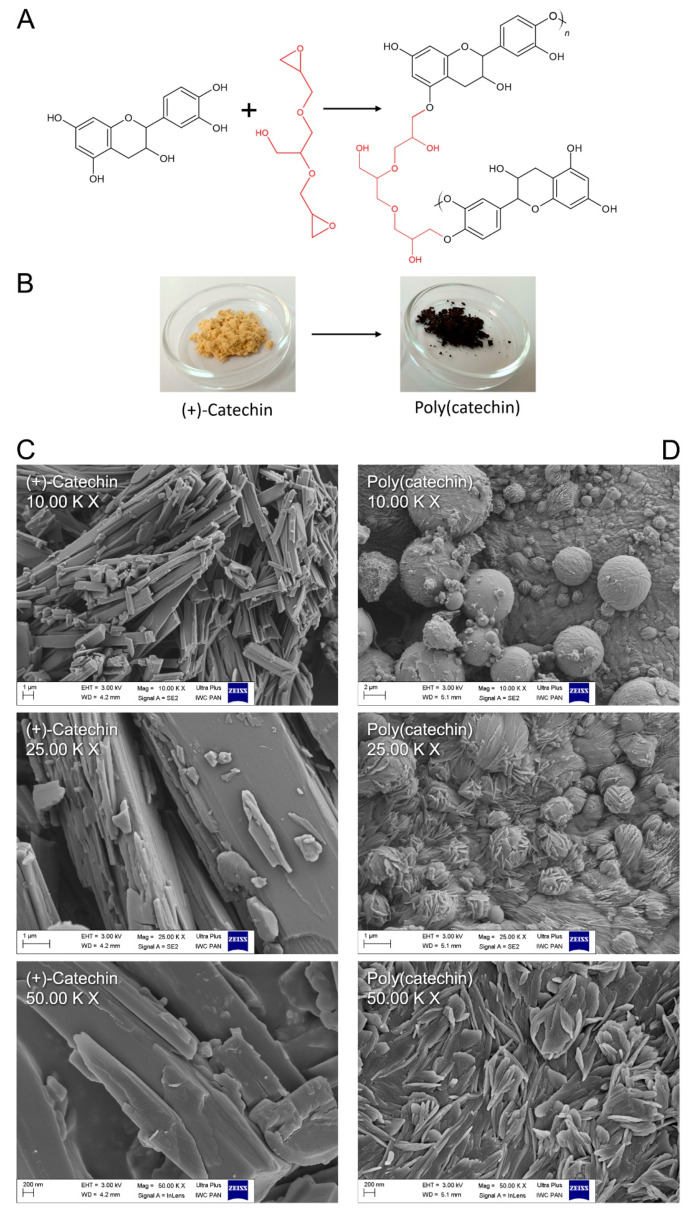
Scheme of the polymerization reaction of (+)-catechin with cross-linking compound (**A**); visual color change of (+)-catechin during polymerization with GDE (digital camera photos) (**B**); SEM images at 10.00 K X, 25.00 K X and 50.00 K X magnification of (+)-catechin (**C**) and poly(catechin) (**D**).

In Figure 1A, the mechanism of catechin cross-linking with GDE is proposed. The epoxy groups in GDE can readily react with the phenol OH groups in (+)-catechin, generating polymeric particles. The reaction was accompanied by a color change of the catechin powder from slightly orange to black as shown in the photos in Figure 1B. In addition to the color change, there was also some change in catechin powder morphology relative to poly(catechin).

Based on the SEM images (Figure 1C,D), the morphology of the samples was assessed. The (+)-catechin was characterized by a needle-shaped structure (Figure 1C). As a result of the cross-linking reaction, (+) catechins were linked together by GDE. In the SEM pictures of poly(catechin) (Figure 1D), unlike the needle-shaped monomer, ball-shaped structures covered with fine needles were visible. The morphology of poly(catechin) resembled knots of wool. The ball-shaped structures could correspond to the GDE crosslinker. The needles were particles of catechin, however, and as a result of polymerization, they became fragmented.

The achieved poly(catechin) powder was prepared for liquid NMR analysis, however it was only partially soluble in water, DMF and DMSO, which made liquid NMR analysis impossible. As test results, only signals corresponding to the solvents were obtained (deuterated water 4.8 ppm and DMSO 2.5 ppm; 3.3–4.8 ppm). The ^1^H NMR spectra of poly(catechin) in deuterated water and DMSO are shown in supplementary materials (Figure S1). Limited solubility may indicate a strong cross-linking of the polymeric flavonoid, and also makes the structure analysis much more difficult.

**Figure 2:**
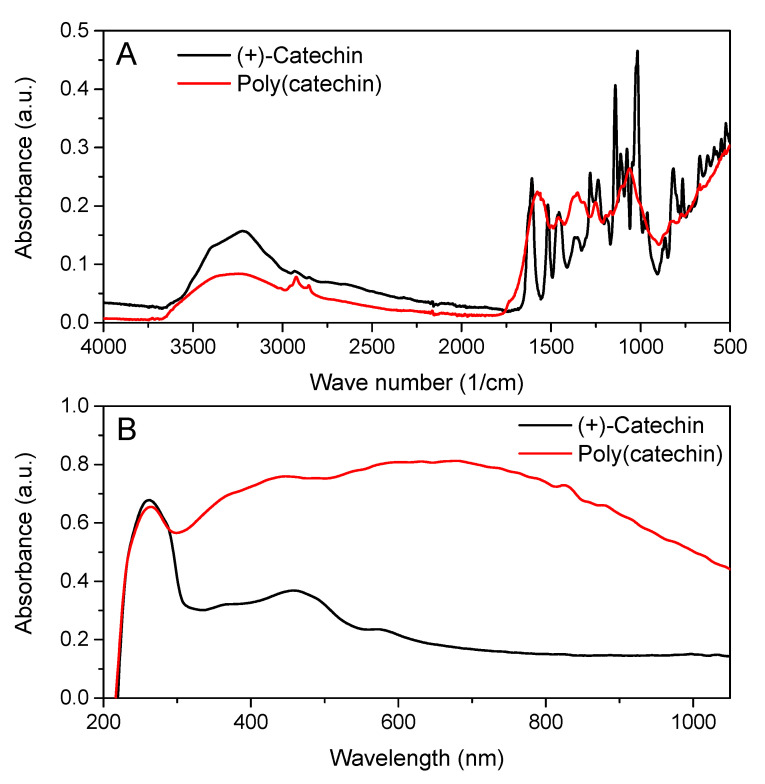
FTIR spectra (**A**) and UV-Vis spectra (**B**) of (+)-catechin and poly(catechin) powders.

Particle formation via the epoxy crosslinking reaction of (+) catechin with GDE was confirmed by FTIR spectroscopy as shown in Figure 2A. As can be seen from Figure 2A, the infrared spectrum of poly(catechin) powder differed from the reference (+)-catechin spectrum, which indicated that a compound with a different structure was obtained from the (+)-catechin monomer.

Infrared spectroscopy confirmed the structure of the polymeric catechin. According to the literature data [26,27] on the polymerization with the cross-linking compound of other flavonoids, such as quercetin and rutin, the following bands present in the spectrum of poly (catechins) were characteristic of the polymeric form of flavonoids: 1370–1250 cm^−1^ - aryl stretching vibrations, 3700–3000 cm^−1^ - wide bands corresponding to the formation of free OH derived from GDE, 1061 cm^−1^ - C-CO-C in ketones, and 750– 790 cm^−1^ as well as 800– 900 cm^−1^ - epoxies (from GDE). In addition, the spectrum showed the peaks characteristic of the functional groups present in flavonoids: 2930– 2920 cm^−1^ - Ar-CH_3_ (more intense than with catechin) and also 1560– 1570 cm^−1^ and 1450–1500 cm^−1^ [32]. The appearance of the poly(catechin) bands characteristic for the polymeric forms of flavonoids in the spectrum indicated the cross-linking reaction of (+)-catechin and the obtaining of a macromolecular/polymeric compound.

Based on the FTIR spectra, the degree of (+)-catechin to poly(catechin) conversion was determined. The peak between 1450 and 1500 cm^−1^, corresponding to the aromatic ring vibration, was used for the calculations. The peak at 1455 cm^−1^ did not change after the polymerization reaction and therefore it can be used as an internal reference [33,34]. Moreover, the peak at 1360 cm^−1^, typical for the aryl stretching vibrations was used to calculate the degree of conversion. The appearance of new aryl bonds was characteristic for polymerization of catechin, as well as for other poly(flavonoids) [26,27]. The height of the peaks was measured in centimetres from the baseline to the maximum point of the absorbance band. The degree of conversion of (+)-catechin to poly(catechin) was calculated according to the Equation (4):

(4) $$DC\;\left(\%\right)=\;\left(\frac{\frac{h_{1360}}{h_{1455}}\;poly\left(catechin\right)}{\frac{h_{1360}}{h_{1455}}\;catechin}-1\right)\times 100\%$$

where *h_1360_* is the height of the band at 1360 cm^−1^, and *h_1455_* is the height of the band at 1455 cm^−1^.

The degree of conversion of (+)-catechin to poly(catechin) was 90%. The high value of the degree of conversion may indicate a good efficiency of the catechin cross-linking reaction.

The UV-Vis spectroscopy also showed a change in catechin structure after the reaction with GDE. Figure 2B shows the UV-Vis spectra of (+)-catechin and poly(catechin) powders. The (+)-catechin spectrum had two characteristic peaks with maxima at 250 and 460 nm. Poly(catechin) powders were also characterized by two peaks - with a maximum at 250 nm and a broad peak between 300 and 900 nm. According to the literature, a broad peak between 300 nm and 550 nm is specific for oligomeric form of catechins, obtained by enzymatic oligomerization [35]. Additionally, the broad peak between 300 and 750 nm was typical for the poly(catechin) obtained in the photopolymerization reaction [36].

**Figure 3:**
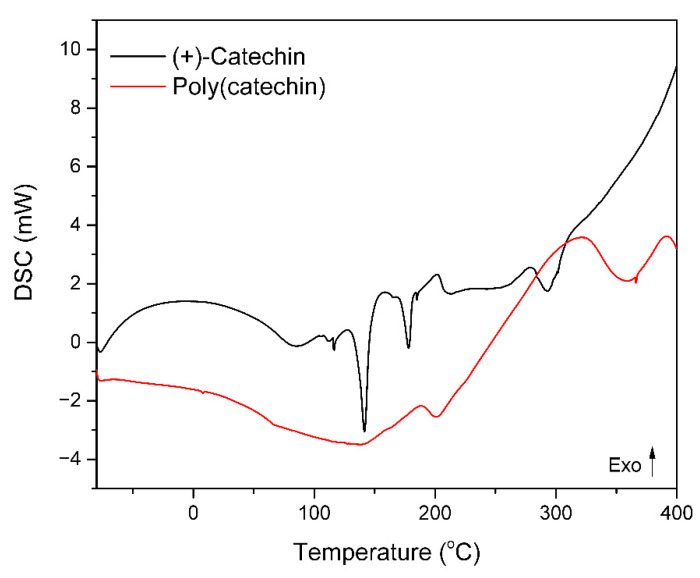
DSC thermograms of (+)-catechin and poly(catechin).

Poly(catechin) powder was subjected to differential scanning calorimetry DSC. The samples were heated from −80 to 400 °C at a rate of 10 °C/min in an air atmosphere. For comparison, differential scanning calorimetry of the reference (+)-catechin was performed. The results are shown in the thermogram in Figure 3 and in Table 1.

The (+)-catechin thermogram showed two endothermic peaks corresponding to the melting of the sample and exothermic peak related to oxidation and degradation of flavonoid. On the thermogram of poly(catechin) were also found endtothermic peak corresponding to the melting of the material and an exothermic peak of oxidation associated with the decomposition of the poly(catechin). Both tested samples did not have the glass transition temperature T_g_.

The poly(catechin) had a lower melting point than the monomeric flavonoid. This may be due to the addition of the GDE cross-linker, which may lower the T_m_. The enthalpy of melting of poly(catechin) (173.5 ± 1.4 J/g) was about 2.5 times higher than that of melting of catechin (49.4 + 20.5 = 69.9 ± 1.4 J/g). Moreover, the poly(catechin) had a higher final oxidation temperature T_o_ (by 65.5 °C) and a higher enthalpy of oxidation ΔH_o_ (about 20 times) then (+)-catechin. Thus, the polymeric form of catechin showed greater resistance to oxidation than the monomeric (+)-catechin. Due to the cross-linked structure of poly(catechin), oxidative processes proceed slower than in monomeric flavonoid. In the monomeric structure of the (+)-catechin there may be more unbound functional groups that react with oxygen during oxidation. In poly(catechin) these groups are linked by network nodes and their oxidation may be hindered and limited.

**Figure 4:**
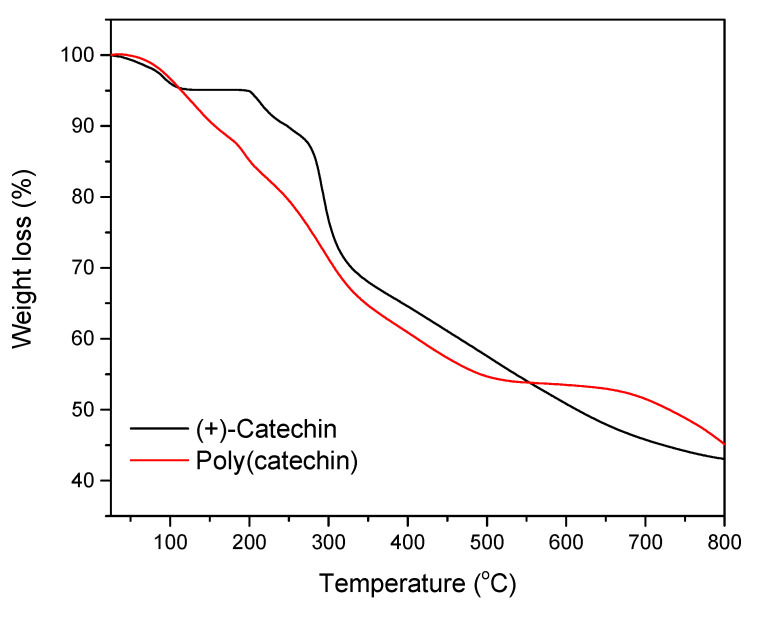
Thermal decomposition (TG) curves of (+)-catechin and poly(catechin).

In the next step, the thermal stability of the flavonoid and the poly(flavonoid) was determined using thermogravimetry. The results of the TG analysis are shown in Figure 4 and Table 2. 

The decomposition of (+)-catechin was in two stages (Figure 4). The first stage took place at 200 °C and was accompanied by a weight loss of 4.9%. The second stage of catechin decomposition took place in the temperature range of 282– 327 ± 0.7 °C, for which a weight loss of 52% was noted. Poly(catechin) also decomposed in two stages. The first stage of decomposition occurred around 200 °C, and the weight loss was 6.7%. The second stage of decomposition followed in the temperature range of 230–360 ± 0.7 °C. The second stage was accompanied by a sample weight loss of only 22.3%.

Table 2 shows the values of T10, T50, and T55 for the samples analyzed, where T10, T50, and T55 refer to the loss of 10%, 50%, and 50%, respectively, of the initial mass of the material as a function of temperature. The T10, T50 and T55 were determined because further weight loss of the samples was not visible at the measurement conditions (25–800 °C). The degradation of poly(catechin) started at a lower temperature than that of catechin (T10 poly(catechin) = 156 ± 0.7 °C, T10 (+)-catechin = 248 ± 0.7 °C). This may have been due to the addition of the GDE cross-linker, which could lower the T10 value. The temperature of the half-decomposition of poly(catechin) T50 was by 117 ± 0.7 °C higher than that of the monomeric (+)-catechin, which indicated higher thermal stability of the polymeric form of (+)-catechin.

The temperature of half-decomposition of poly(catechin), higher by 117 ± 0.7 °C, and about two times lower weight loss of poly(catechin) during the second stage of decomposition of the compound, testified to higher thermal stability of the polymeric form of (+)-catechin. The cross-linked structure of poly(catechin) can limit heat ingress into the molecules and improves thermal stability. Moreover, in the polymeric (+)-catechin there may be fewer unbound functional groups that are less thermally resistant than those linked by nodes.

In accordance with the literature data [37], the complex aromatic structure of natural condensed catechins— tannins lead to high thermal resistance. It has been described that the decomposition of *Acacia dealbata* tannin was almost complete at a temperature of 600 °C, the remaining weight of tannin was approximately 44%, so the weight loss of the sample was 56%. The weight loss of poly(catechin) at 600 °C was about 47% and of the reference catechin was about 49%. Comparing the obtained results with the literature on the condensed tannin, it should be stated that both (+)-catechin and poly(catechin) at 600 °C had lower thermal stability than natural tannin.

**Figure 5:**
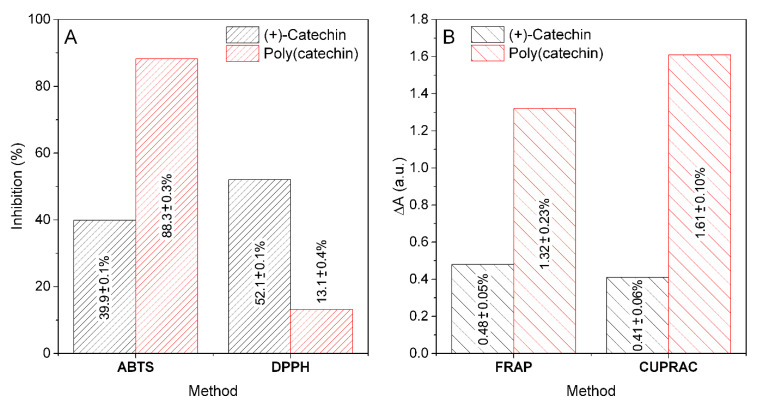
Ability of 0.1 mg/mL (+)-catechin and poly(catechin) to reduce free radicals ABTS and DPPH (**A**) and for reduce of iron and cupric ions measured by FRAP and CUPRAC methods (**B**).

Figure 5 shows the activities of (+)-catechin and poly(catechin) for the reduction of free radicals ABTS and DPPH and for the reduction of transition metal ions - iron (FRAP) and copper (CUPRAC). (+)-Catechin and poly(catechin) solutions at a concentration of 0.1 mg/mL in distilled water were prepared for the analyzes. Catechin was highly soluble and poly(catechin) only partially. The limited solubility of the polymeric (+)-catechin could have influenced the research results. In addition, poly(catechin) solutions were characterized by an intense, dark color, which could also affect the results of spectrophotometric colorimetric methods.

The applied ABTS and DPPH methods are based on the reactions of quenching synthetic free radicals. In such methods, the colored active radical is reduced by the antioxidant present in the test sample to a colorless product. The ABTS method enables the determination of hydrophobic and hydrophilic antioxidants. The analogous DPPH method is very sensitive and serves only for the analysis of hydrophobic compounds. The polymeric form of (+)-catechin showed a very high antioxidant activity towards the reduction of ABTS radicals (88.3 ± 0.3%; TEAC 662.3 ± 3.1 mmolT/100 g). The activity for reducing ABTS radicals by poly(catechin) was much greater (around 2.2 times) than that of the monomeric form (39.9 ± 0.1%; TEAC 331.8 ± 1.2 mmolT/100 g). In contrast to the ABTS method, in the DPPH method, poly(catechin) was characterized by lower antiradical activity (about 3.9 times; 13.0 ± 0.4%; TEAC 477.6 ± 2.5 mmolT/100 g) than (+)-catechin (52.1 ± 0.1%; TEAC 492.6 ± 1.9 mmolT/100 g). The results of ABTS and DPPH tests may indicate a better affinity of poly(catechin) to the ABTS method intended for the determination of hydrophilic and hydrophobic compounds, in contrast to the DPPH method - intended for the analysis of only hydrophobic compounds. Sahiner showed similar results in the determination of DPPH for rutin and poly(rutin) [27]. The scavenging capacity of DPPH free radicals by poly(rutin) particles is lower compared to rutin monomer and α-tocopherol. The poly(rutin) particles still have phenolic groups on their surfaces and have the ability to annihilate DPPH radicals. According to author, this difference could be attributed to the bigger size of particles of poly(rutin) and the lesser number of OH functional groups in comparison to rutin. Active groups that scavenged DPPH free radicals could be linked by network nodes during polymerization, as a result of which their number decreased, and thus the ability to scavenge DPPH radicals decreased.

The second group of spectrophotometric methods that were used to analyze (+)-catechin and poly(catechin) were methods based on the reduction of multivalent metal ions. The poly(catechin) obtained as a result of the reaction with the cross-linker was characterized by a better ability to reduce iron ions (FRAP assay, about 2.7 times) and copper (CUPRAC test, around 3.9 times). The polymerization of (+)-catechin had a positive effect on the improvement of these properties. Polyvalent metal ions can catalyze the aging processes, therefore the ability to reduce and chelate such ions is a very important property for potentially stabilizers.

Samples of (+)-catechin and poly(catechin) were tested for antibacterial and antifungal activity. The test results are summarized in Table 3.

After 24 h of incubation, an increase in the number of all organisms was observed in the control medium without polyphenols. An increase in the number of *Escherichia coli* bacterial cells by about 1 to 1.8 logarithm was recorded in cultures with all materials, which meant that the samples did not show any antibacterial activity against this organism. The increase in the number of *Staphylococcus aureus* bacterial cells in the culture for poly(catechin) did not exceed 0.5 logarithm, which means that the poly(catechin) sample showed bacteriostatic activity against this organism. The reference (+)-catechin showed no such activity. The increase in the number of *Bacillus subtilis* bacterial cells was recorded in cultures with all materials at similar levels, which means that the samples showed no antibacterial activity against this bacterial cell.

The increase in the number of *Candida albicans* yeast cells was recorded in the cultures with all samples. (+)-Catechin and poly(catechin) showed no antimicrobial activity against this organism. The number of *Aspergillus niger* mold cells after 24 h increased by more than one row in the polyphenol-free control only. In the remaining samples, containing (+)-catechin and poly(catechin), a decrease in the number of cells was noted in the cultures with both compounds, which meant that they showed antifungal activity. (+)-Catechin and poly (catechin) showed comparable antifungal activity against *Aspergillus niger*.

In conclusion, the polymeric form of (+)-catechin showed antimicrobial activity against *Staphylococcus aureus* cells. Such activity was not found for the (+)-catechin monomer. Similar results were observed by Sahiner for poly(quercetin) obtained in the reaction with GDE [26]. The author found that poly(quercetin) had a stronger antibacterial effect than quercetin (concentration 0.01 g/mL) on the tested *E. coli* ATCC 8739, *S. aureus* ATCC 25323, and *B. subtilis* ATCC 6633 strains.

In the case of antifungal activity, both (+)-catechin and poly(catechin) were active against *Aspergillus niger* mold cells. Polyphenols do not completely inhibit the growth of microorganisms, but they prolong the adaptation phase, which was clearly visible in the case of organisms that grow longer, such as molds. The obtained results and the lack of antimicrobial activity may also result from the low solubility of the preparations in water and thus the difficult penetration into the cells.

## 4. Conclusions

As a result of the cross-linking reaction with GDE, the polymeric form of (+)-catechin was obtained. The limited solubility of poly(catechin) powder made it difficult to determine the structure of the compound. However, FTIR analysis indicated the presence of polymeric bonds characteristic of poly (flavonoids). Moreover, the UV-Vis spectra also had peaks corresponding to the polymeric forms of poly(catechins). The polymeric form of (+)-catechin was characterized by greater resistance to oxidation than the monomer (DSC analysis). Moreover, on the basis of the determination of TG it was found that poly(catechin) has a higher thermal stability than (+)-catechin, however, this stability at 600 °C is not as high as that of natural condensed tannin. Analysis of the antioxidant activity showed that the polymeric (+)-catechin had better activity for reducing ABTS free radicals and worse for reducing DPPH. Moreover, polymerization of (+)-catechin increased the ability to reduce polyvalent metal ions. Poly(catechin) showed antibacterial activity against *Staphylococcus aureus*, stronger than (+)-catechin, as well as antifungal activity against *Aspergillus niger*, comparable to the activity of the monomer. Thanks to good resistance to oxidation, high thermal stability, and great ability to reduce metal ions, poly(catechin) can potentially be stabilizers, e.g., for polymeric materials and environmentally friendly materials. Additionally, due to its antimicrobial properties, poly(catechin) can be proposed as a natural functional additive, e.g., for polymeric active packaging.

## Figures and Tables

**Table 1:** DSC analysis of (+)-catechin and poly(catechin).

Sample	T_g_ [°C]	ΔH_m_ [J/g]	T_m_ [°C]	ΔH_o_ [J/g]	T_o_ [°C]
(+)-Catechin	a.u.	49.4 20.5	134.3 171.4	13.6	286.3 (endset)
Poly(catechin)	a.u.	173.5	126.5	274.6	351.8 (endset)

T_g_ - glass transition temperature, ΔH_m_ - melting enthalpy, T_m_ - melting point, ΔH_o_ - oxidation and degradation enthalpy, T_o_ - oxidation and degradation temperature. Standard deviations: temperature ± 0.8 °C; enthalpy ± 1.4 J/g.

**Table 2:** T10, T50, and T55 of (+)-catechin and poly(catechin). Standard deviation: temperature ± 0.7 °C.

Sample	T10 (°C)	T50 (°C)	T55 (°C)
(+)-Catechin	248	613	719
Poly(catechin)	156	730	800

**Table 3:** Antibacterial and antifungal activity of (+)-catechin and poly(catechin).

Sample	The Number of Microorganisms [cfu/cm^2^]		Log of the Number of Microorganisms		D
	t = 0	t = 24	t = 0	t = 24	
***Escherichia coli***					
**Control medium**	1.67 ± 0.21 × 10^6^	9.57 ± 1.56 × 10^7^	6.23 ± 0.05	7.98 ± 0.07	1.75
**(+)-Catechin**		1.63 ± 0.25 × 10^7^		7.20 ± 0.07	0.97
**Poly(catechin)**		1.90 ± 0.29 × 10^7^		7.28 ± 0.05	1.05
***Staphylococcus aureus***					
**Control medium**	6.53 ± 0.25 × 10^5^	1.13 ± 0.13 × 10^8^	5.81 ± 0.03	8.04 ± 0.10	2.23
**(+)-Catechin**		1.63 ± 0.08 × 10^7^		7.20 ± 0.06	1.39
**Poly(catechin)**		1.33 ± 0.30 × 10^6^		6.11 ± 0.10	0.30
***Bacillus subtilis***					
**Control medium**	1.33 ± 0.25 × 10^6^	1.63 ± 0.21 × 10^7^	6.11 ± 0.08	7.20 ± 0.06	1.09
**(+)-Catechin**		1.83 ± 0.21 × 10^7^		7.26 ± 0.05	1.14
**Poly(catechin)**		9.60 ± 0.40 × 10^6^		6.98 ± 0.02	0.87
***Candida albicans***					
**Control medium**	1.87 ± 0.38 × 10^5^	3.74 ± 1.47 × 10^6^	5.28 ± 0.08	6.57 ± 0.18	1.29
**(+)-Catechin**		1.83 ± 0.21 × 10^6^		6.26 ± 0.05	0.98
**Poly(catechin)**		1.50 ± 0.20 × 10^6^		6.18 ± 0.06	0.90
***Aspergillus niger***					
**Control medium**	1.33 ± 0.40 × 10^4^	2.37 ± 0.42 × 10^5^	4.11 ± 0.14	5.38 ± 0.08	1.27
**(+)-Catechin**		4.17 ± 0.55 × 10^2^		2.62 ± 0.06	−1.49
**Poly(catechin)**		6.53 ± 0.31 × 10^2^		2.81 ± 0.02	−1.30

## Data Availability

Not applicable.

## Supplementary Materials

The following are available online at https://www.mdpi.com/2218-273X/11/1/50/s1, Figure S1: ^1^H NMR spectra of poly(catechin) in deuterated water and in DMSO.
