# Supplementary material for: Thermally Stable and Antimicrobial Active Poly(Catechin) Obtained by Reaction with a Cross-Linking Agent
Source: Biomolecules. 2020 Dec 31;11(1):50. doi: 10.3390/biom11010050 (PMC7823284; doi:10.3390/biom11010050)
Supplement: Supplementary file 1 [file biomolecules-11-00050-s001.pdf]

biomolecules

Supplementary Materials

Thermally stable and antimicrobial active poly(catechin) obtained by reaction with a cross-linking agent

Malgorzata Latos-Brozio [1], Anna Masek [1,*] and Malgorzata Piotrowska [2]

[1] Faculty of Chemistry, Institute of Polymer and Dye Technology, Lodz University of Technology, Stefanowskiego 12/16, 90-924 Lodz, Poland; malgorzata.latos@p.lodz.pl
[2] Faculty of Biotechnology and Food Sciences, Institute of Fermentation Technology and Microbiology Lodz University of Technology, Wólczańska 71/173, 90-924 Lodz, Poland; malgorzata.piotrowska@p.lodz.pl
* Correspondence: anna.masek@p.lodz.pl (A.M.)

Figure S1. ^{1}H NMR spectra of poly(catechin) in deuterated water and in DMSO.

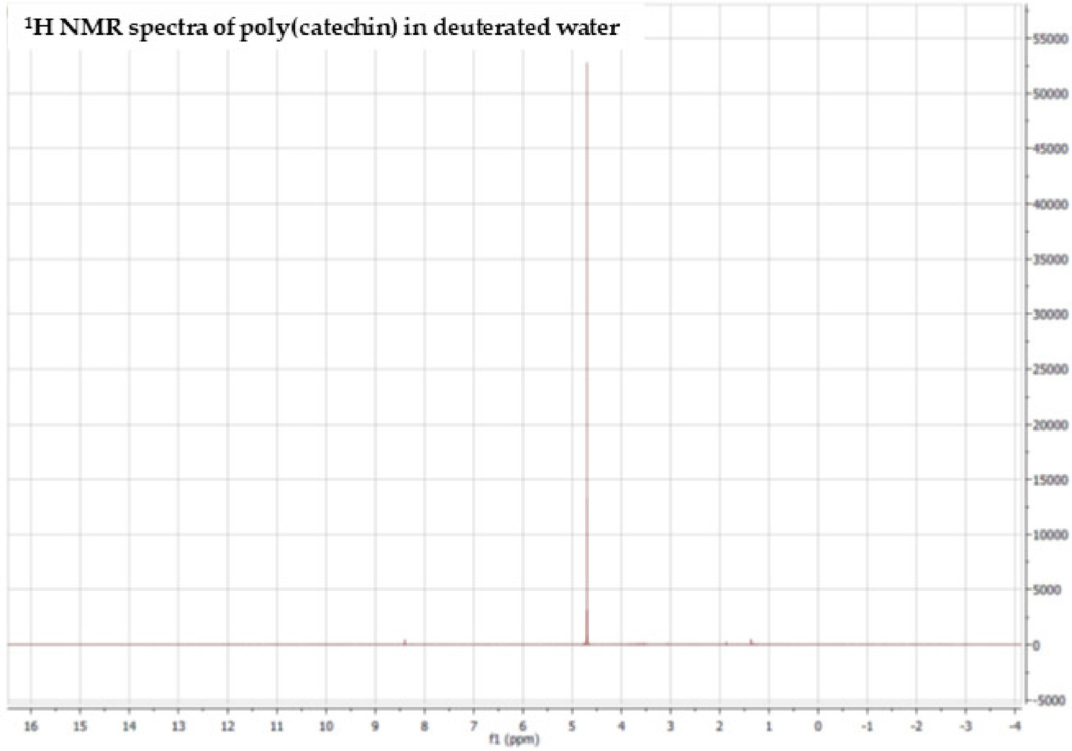

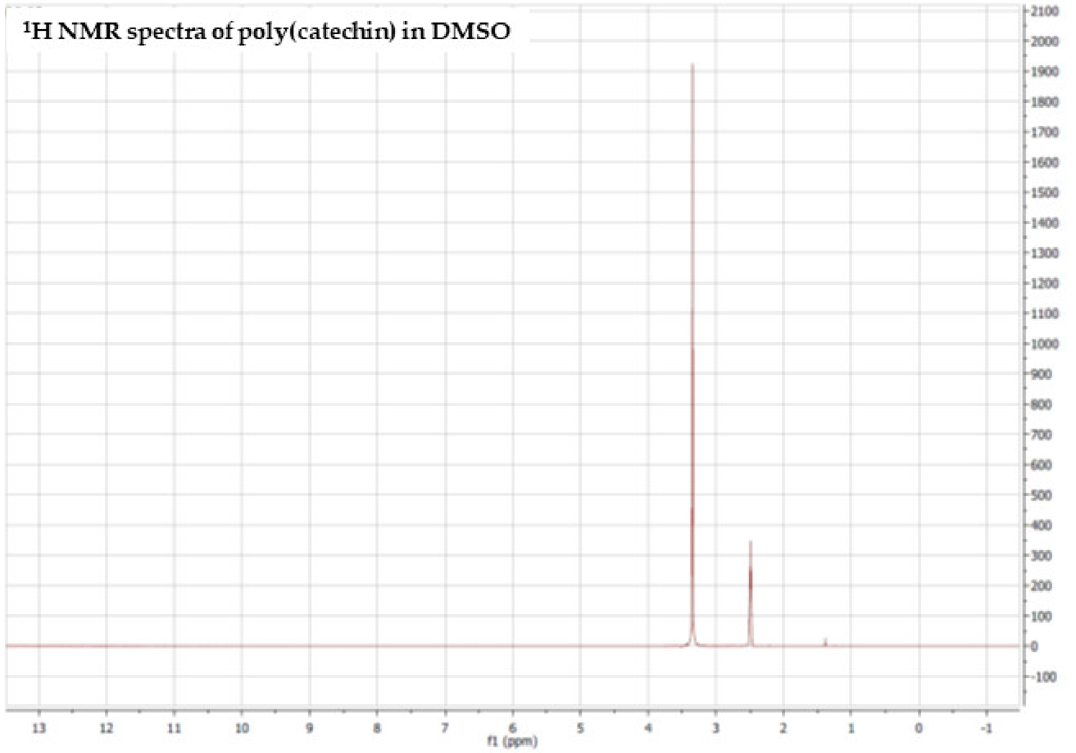

Figure S1. ^{1}H NMR spectra of poly(catechin) in deuterated water and in DMSO.
